# EEG Power Spectral Analysis of Abnormal Cortical Activations During REM/NREM Sleep in Obstructive Sleep Apnea

**DOI:** 10.3389/fneur.2021.643855

**Published:** 2021-02-26

**Authors:** Shuling Liu, Jiucheng Shen, Yezhou Li, Jing Wang, Jianhua Wang, Juan Xu, Qiaojun Wang, Rui Chen

**Affiliations:** ^1^Department of Respiratory Medicine, Sleep Center, The Second Affiliated Hospital of Soochow University, Soochow University, Suzhou, China; ^2^Department of Respiratory Medicine, The Second Affiliated Hospital of Soochow University, Suzhou, China; ^3^School of Medicine, The University of Manchester, Manchester, United Kingdom; ^4^Sleep Center, The Second Affiliated Hospital of Soochow University, Suzhou, China

**Keywords:** obstructive sleep apnea, polysomnography, REM and NREM sleep, power spectral analysis, normalized EEG power

## Abstract

**Objective:** To characterize electroencephalogram (EEG) power in different frequency bands during rapid eye movement (REM) sleep and non-rapid eye movement (NREM) sleep in patients with obstructive sleep apnea (OSA).

**Methods:** Retrospective data on 151 patients were collected and divided into three groups: primary snoring group (AHI < 5/h), mild-moderate OSA group (6 ≤ AHI < 30/h), and severe OSA group (AHI ≥ 30/h). EEG recordings in the frontal, central, and occipital regions were extracted from both REM and NREM sleep, to compute the normalized spectral power densities in the delta, theta, alpha, sigma, beta, and gamma frequency bands, using Fast Fourier Transform. Correlations between the computed EEG power and PSG parameters were analyzed.

**Results:** In NREM sleep, elevated normalized power spectral density (PSD) in the delta band was observed in the severe OSA group compared to the other two groups. In contrast, the PSD of the other frequency bands showed a corresponding decrease in the severe OSA group. In REM sleep, similar changes were observed in the frontal region. Delta band PSD was positively correlated with Apnea Hypopnea Index (AHI) (*r* = 0.33), longest time of apnea, oxygen desaturation index (ODI) (*r* = 0.34), percent sleep time below 90% SaO_2_ (T90%) (*r* = 0.30), Arousal Index (ArI) (*r* = 0.29), and negatively correlated with N3%, minimum oxygen saturation (minSaO_2_).

**Conclusion:** Our findings provide neurophysiological evidence for pathological cortical activation during REM/NREM sleep, which may be associated with the arousals and cognitive impairments in OSA. The technique of power spectral analysis could prove a potentially useful tool in complementing traditional PSG parameters in assessing disease burden to guide therapeutic decisions.

## Introduction

Obstructive Sleep Apnea is one of the most common types of sleep-disordered breathing (SDB) (1–3). It is characterized by recurrent upper airway collapse during sleep, often leading to repeated awakening, sleep fragmentation, and reduced slow-wave sleep (4, 5). Increasingly, OSA is regarded as a systemic disease that impacts multiple organs of the metabolic, cardio- and cerebrovascular systems, elevating all-cause mortality (6, 7).

Sleep fragmentation and frequent awakening in OSA patients lead to daytime sleepiness, reduced working and learning efficiency, cognitive dysfunction, as well as increased risks of diseases such as hypertension, stroke, diabetes, and thus reduced overall quality of life (8). At present, several major problems remain unsolved regarding the pathophysiology as well as the clinical diagnosis and treatment of OSA: (1) clinicians face significant difficulties in evaluating the disease burden of OSA due to its highly heterogeneous clinical manifestations (9), with symptoms ranging from morning fatigue to memory impairment (see Table 1 for the lack of inter-group differences in reported symptoms); (2) Polysomnography is the gold standard for OSA diagnosis (10, 11), but the current measures of disease severity (AHI, ODI, the proportions of each sleep stage) do not strongly and consistently relate to comorbidity and mortality risks (12–14); and (3) the current suite of clinical assessment questionnaires on sleep quality, daytime sleepiness, and cognitive function, viz. the Epworth Sleepiness Scale (ESS), Pittsburgh Sleep Quality Index (PSQI), Mini-Mental State Examination and Montreal Cognitive Assessment (MoCA), tends to be subjective, and lacks specificity and sensitivity in the early stages of the disease. Therefore, current clinical practice calls for better biomarkers that might objectively capture the disease burden faced by patients to better guide therapeutic decisions.

**Table 1:** Demographic and clinical characteristics in the primary snoring group, mild-moderate OSA group, and Severe OSA group.

**Parameter**	**Primary snoring**	**Mild-moderate OSA**	**Severe OSA**	***P*-value**
	**(*N =* 40)**	**(*N =* 48)**	**(*N =* 63)**	
Age, years	36 (31, 39)	37 (32, 41)	42 (36, 49)^a^^,^ ^b^	<0.01
BMI, kg/m^2^	25.4 (23.7, 26.8)	26.2 (24.6, 27.8)	28.5 (26.1, 31.0)^a^^,^ ^b^	<0.01
**COMMON SYMPTOMS**				
Nocturia	22 (51%)	31 (65%)	44 (70%)	0.14
Leg movements	12 (28%)	9 (19%)	16 (25%)	0.62
Dreaming	30 (70%)	31 (65%)	46 (73%)	0.65
Nightmare	3 (7.0%)	7 (15%)	6 (9.5%)	0.52
Morning fatigue	23 (53%)	26 (54%)	40 (63%)	0.51
Morning headache	5 (12%)	5 (10%)	12 (19%)	0.46
Daytime sleepiness	22 (51%)	29 (60%)	46 (73%)	0.07
Poor memory	17 (40%)	19 (40%)	16 (25%)	0.23
Insomnia	5 (12%)	3 (6.2%)	7 (11%)	0.62
Difficulty falling asleep	5 (12%)	3 (6.2%)	4 (6.3%)	0.68
Early awakening	13 (30%)	8 (17%)	16 (25%)	0.31
**COMORBIDITIES**				
Diabetes	0	1 (2.10%)	1 (1.60%)	0.68
Coronary artery disease	0 (0%)	2 (4.2%)	1 (1.6%)	0.53
Hypertension	9 (21%)	6 (12%)	25 (40%)^a^	<0.01
Arrythmia	4 (9.3%)	3 (6.2%)	5 (7.9%)	0.93
Asthma	1 (2.3%)	2 (4.2%)	2 (3.2%)	0.96
**QUESTIONNAIRES**				
PSQI score	6 (5, 9)	5 (4, 8)	6 (5, 7.5)	0.38
ESS score	6 (4, 10)	7 (3, 9)	9 (7, 13)^b^	<0.01

*Data are presented as mean ± SD, count (percentage), or median (interquartile range) unless otherwise indicated*.

*BMI, body mass index; PSQI, Pittsburgh Sleep Quality Index; ESS, Epworth Sleepiness Scale*.

a Compared with Primary snoring group, P < 0.05;

b *Compared with Mild-moderate OSA group, P < 0.05*.

Power spectral analysis (PSA) provides a more objective alternative perspective for the analysis of EEG signals by leveraging Fast Fourier Transform (FFT) to convert time-domain EEG signals (amplitude vs. time) to frequency-domain signals (amplitude vs. frequency). This technique reveals additional information about brain activities during sleep, which might otherwise be hidden to clinicians examining the plain EEG signals on PSG tracings. PSA quantifies the amplitude of each frequency component band that constitutes the EEG waveform in the form of a power spectrum, with amplitude plotted against frequency (15). The literature has much on the physiological significance of each frequency band during different sleep stages: (1) delta activities (1–4 Hz) is generally considered to be an indicator of sleep depth and homeostasis during NREM sleep (16, 17); (2) theta activities (4–8 Hz) in the frontal region serve as a marker of emotional memory consolidation (18, 19); (3) alpha activities (8–12 Hz) are suggested to be a cortical signature of visual perception and mental imagery (20), and a reflection of neuronal activities, with low alpha levels indicating a state of excitation and vice versa (21, 22); (4) sigma activities (12–15 Hz) during N2 sleep serve as a proxy for the sleep spindle and is shown to facilitate learning and memory consolidation (23) and protect sleep from external disturbances (24, 25); and (5) high-frequency activities in the beta (15–30 Hz) and gamma (30–40 Hz) ranges are associated with arousals (24, 26, 27). As such, PSA enables a closer examination of the relative changes in prominence of each frequency band in OSA patients, to reveal the functional significance that underlies the change in EEG patterns.

In this study, we applied the PSA technique to analyze the EEG power spectral distribution variations between OSA patients and primary snoring patients during NREM and REM sleep. Moreover, clinical variables were examined for their correlation with the observed between-group EEG power differences, so as to reveal pathological manifestations of the underlying EEG changes and the clinical significance thereof.

## Method

### Participants

**Figure 1:**
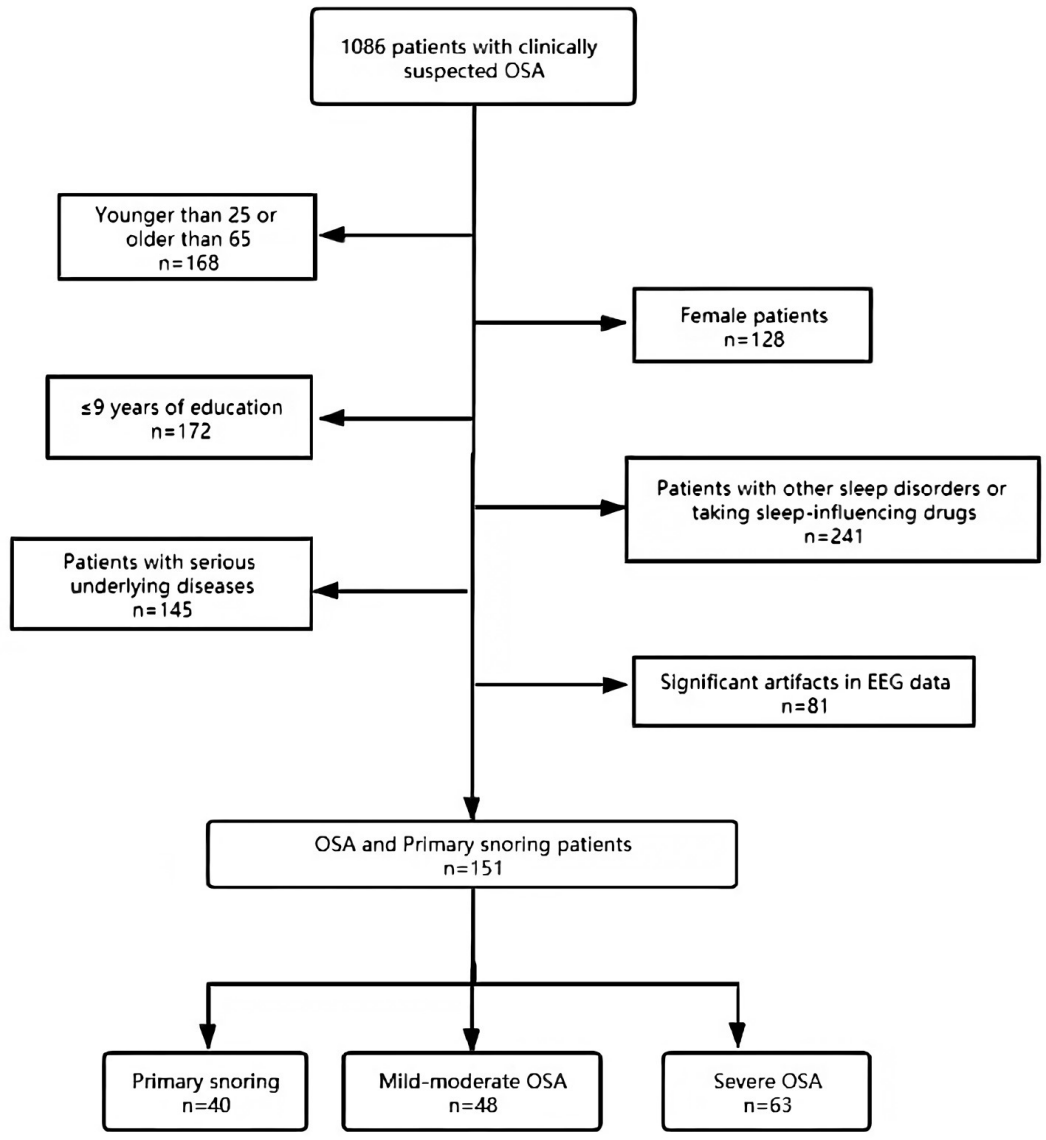
Flow chart showing the patient selection process leading to the final cohort.

A retrospective cohort study was carried out with a selected subset of data from 1,086 patients received with complaints of snoring and a clinical suspicion of OSA at the Sleep Center of the Second Affiliated Hospital of Soochow University from January 2018 to June 2020. In view of previous studies that have shown gender differences in various sleep parameters (28), this study focused primarily on male patients. The patients were between 25 and 65 years old and have received >9 years of education. Individuals taking medications in the last 3 months that are known to impact sleep profiles (e.g., hypnotics, benzodiazepines) or suffering from other sleep disorders (insomnia, central sleep apnea, rapid eye movement sleep behavioral disorder, restless legs syndrome, narcoleptic spectral disorder, periodic limb movement disorder) or significant artifacts in EEG date were excluded. Individuals with serious lung, kidney, liver, or brain diseases were also excluded. Clinical data and PSG studies from 151 subjects were eventually selected and divided into three groups, according to standards recommended by the Chinese Medical Association (29): primary snoring (snoring with AHI <5 events/h; *N* = 40), mild-moderate OSA (5 ≤ AHI ≤ 30 events/h; *N* = 48), and severe OSA (AHI > 30 events/h; *N* = 63) (see Figure 1 for a flow chart of the subject selection process). Epworth Sleepiness Scale (ESS) and the Pittsburgh Sleep Quality Index (PSQI) were administered to all patients to evaluate their sleep quality and the potential presence of excessive daytime sleepiness (30, 31). All subjects completed the questionnaires in a quiet and isolated room. The participants in the study gave informed consent, and the study protocol was approved by the Research Ethics Committee of the Second Affiliated Hospital of Soochow University, Suzhou, China (Batch number: JD-LK-2018-006-01).

### Polysomnography and Sleep Analysis

The participants underwent overnight, supervised, laboratory-based video polysomnography. The PSG recorded from 22:00 to 06:00 the next morning. Compumedics Grael multifunctional PSG monitoring system was used for all signal acquisition (Compumedics Company, Australia). Only PSG readings with an effective monitoring time ≥7 h were included for analysis (32). The polysomnographic recordings included eight electroencephalogram (EEG) channels (F3, F4, C3, C4, O1, O2, A1, and A2, placed according to the international 10–20 system), bilateral electrooculograms (EOGs), submental and bilateral anterior tibialis electromyograms (EMGs), electrocardiograms (ECGs), the nasal and oronasal airflow (by using nasal pressure monitor and thermistor), arterial oxygen saturation (via finger pulse oximetry), thoracic and abdominal movements (via inductance plethysmography), and time-synchronized video recordings with audio.

### Sleep Stage Analysis and Scoring

Sleep stages and sleep-related respiratory analysis were scored manually in 30-s epochs by registered technicians according to the AASM scoring criteria (33). The apnea-hypopnea index (AHI) was calculated as the mean number of apneas and hypopneas (with ≥3% desaturation or an arousal) per hour of sleep. The oxygen desaturation index was defined as the mean number of ≥3% desaturation events per hour of sleep. Other recorded parameters included total sleep time (TST), proportions of each sleep stage, minimum arterial oxygen saturation (minSaO_2_), percent sleep time below 90% SaO_2_ (T90%), sleep efficiency, sleep latency, REM latency, and arousal index (ArI).

### EEG Preprocessing

Thirty-second samples were extracted from EEG recordings during both rapid eye movement (REM) sleep and non-rapid eye movement (NREM) sleep. As NREM sleep is a very heterogeneous state consisting of three distinct stages (N1, N2, and N3), in this study, we chose to focus mainly on N2 sleep, the more stable period during sleep. EEG signals were sampled from epochs free from respiratory events to avoid potential interferences. Using the EEGLAB toolbox in MATLAB (Math works, R2013b), raw data were imported at a sampling rate of 512 Hz and down-sampled to 256 Hz, before applying a band-pass filter between 0.1 and 100 Hz and a notch filter at 50 Hz. We used the linked ear electrodes as a reference and segmented the 30-second REM and NREM sleep epochs into 15 2s mini-epochs. Channels with excessive noise, drift, or unstable connection were interpolated using spherical interpolation. Mini-epochs were visually inspected to exclude those with excessive noise or >3 electrodes with artifacts. To identify mini-epochs with eye movements, Independent component analysis (ICA) was performed using the extended infomax algorithm, and identified segments were manually inspected and removed. Lastly, the EEG was inspected visually again and epochs with excessive noise or artifacts were removed. Impedances of all EEG electrodes were kept below 10 kΩ. The investigator who performed the EEG preprocessing was blinded to the diagnosis.

### Power Spectral Analyses

Selected mini-epochs in each EEG electrode (F3, F4, C3, C4, O1, O2) were Hanning-tapered and Fourier transformed by the FFT algorithm (with 50% overlapping windows) to obtain the spectral power density at each frequency. We assessed the following six frequency bands: delta (0.5–4 Hz), theta (4–8 Hz), alpha (8–12 Hz), sigma (12–15 Hz), beta (15–30 Hz), and gamma (30–45 Hz), with each band inclusive of the lower boundary but not the upper boundary. Normalized band power [also known in some other studies as relative EEG power (34), and equivalently percent power (35)] was then calculated by normalizing the total power (0.5–45 Hz) for inter-participant comparisons. That is, the absolute band spectral power measurements of each participant were multiplied by a factor such that all participants share the same total power in the frequency range of interest (0.5–45 Hz) (Normalization was conducted separately for each sleep stage and electrode location). This method thus accounts better for the relative contribution and thus the prominence of each frequency band and reduced the cross-participant variance. Activities between 48 and 52 Hz were excluded from the analysis to remove potential power line interferences. Scalp electrodes were grouped into three regions of interest (ROIs), including the frontal (F3, F4), central (C3, C4), and occipital (O1, O2) regions.

### Statistical Analysis

The statistical analyses were carried out using the SPSS software (version 17.0, SPSS, Inc. Chicago, IL, USA). The measured data are presented as the mean ± standard deviation if normally distributed or otherwise median (with interquartile range in brackets). Normally distributed variables were compared using one-way analysis of variance, and the non-normally distributed variables using the Mann-Whitney *U* test. Categorical variables are presented as the frequency distribution and were compared using the chi-squared test or Fisher's exact test. Partial Spearman correlation analyses were used to evaluate the relationship between the clinical variables and normalized power spectral density in each frequency band, adjusting for patients' age. The *p*-values were adjusted for multiple comparisons using the Benjamini-Hochberg procedure. A false discovery rate of *p* < 0.05 was considered statistically significant.

## Results

### Demographics and Sleep Microarchitecture

An initial cohort of 1,086 patients with complaints of snoring and a clinical suspicion of OSA were identified in the database, from which a subset of 151 patients was selected to form the final cohort (see Figure 1 for details on patient selection). The demographic and clinical characteristics are shown in Table 1. The median age was 38 years. The patients tend to be older, with higher BMI, score higher in ESS, and are at higher risks for hypertension in the severe OSA group than in the other two groups (all *p* < 0.05). We found no significant differences in PSQI score and the prevalence of diabetes, arrhythmia, stroke, coronary artery diseases, and asthma among the three groups. Notably, no significant inter-group differences were found in terms of patient-reported symptoms, including daytime sleepiness, insomnia, and poor memory (all *p* > 0.05). Table 2 summarizes the PSG parameters for all three groups. Increasing OSA severity is associated with higher N1%, ODI, T90%, arousal index and longest time of apnea, lower N3%, and minSaO_2_ (*p* < 0.05 and mostly *p* < 0.01 between any two severity groups).

**Table 2:** PSG parameters in the primary snoring group, mild-moderate OSA group, and Severe OSA group.

**Parameter**	**Primary snoring**	**Mild-moderate OSA**	**Severe OSA**	***P*-value**
	**(*N =* 40)**	**(*N =* 48)**	**(*N =* 63)**	
AHI, n/hr	2.5 (1.4, 4.0)	13.7 (8.3, 19.8)^a^	61.4 (48.2, 75.2)^a^^,^ ^b^	<0.01
TST, min	432.8 (381.8, 465.8)	404.8 (351.8, 454.0)	417.0 (362.8, 488.2)	0.33
Sleep efficiency %	88.7 (79.3, 92.8)	87.6 (72.3, 91.9)	83.1 (74.4, 91.6)	0.68
Sleep latency, min	4.3 (0.3, 8.8)	5.5 (2.3, 16.8)	3.5 (1.0, 13.3)	0.20
REM latency, min	87.0 (66.0, 121.0)	78.3 (58.0, 129.8)	97.8 (76.0, 148.5)	0.12
NREM 1 %	5.9 (3.8, 10.5)	9.0 (6.8, 13.7) ^a^	17.0 (11.3, 30.4)^a^^,^ ^b^	<0.01
NREM 2 %	53.5 (45.2, 57.4)	51.9 (47.2, 56.0)	54.8 (46.8, 63.6)	0.18
NREM 3 %	20.9 (16.6, 24.9)	16.1 (12.3, 21.1)^a^	5.1 (0, 8.65)^a^^,^ ^b^	<0.01
REM sleep%	21.0 (17.6, 25.1)	22.1 (17.3, 25.9)	19.6 (13.6, 23.4)	0.10
ODI	1.5 (0.4, 2.7)	9.0 (4.5, 12.8) ^a^	54.4 (39.2, 70.2) ^a^^,^ ^b^	<0.01
minSaO_2_	90.0 (89.0, 91.5)	84.0 (80.5, 88.5)^a^	69.0 (59.5, 77.5)^a^^,^ ^b^	<0.01
T90%	0 (0, 0.1)	0.7 (0.3, 2.1) ^a^	18.7 (9.0, 44.8)^a^^,^ ^b^	<0.01
ArI	6.3 (3.6, 9.3)	11.3(7.3, 14.0)^a^	41.3 (26.5, 54.0)^a^^,^ ^b^	<0.01
Longest apnea, sec	31.0 (22.0, 44.0)	44.5 (38.0, 57.0)^a^	72.0 (58.0, 88.0)^a^^,^ ^b^	<0.01

*Data are presented as mean ± SD, count (percentage), or median (interquartile range) unless otherwise indicated*.

*AHI, Apnea Hypopnea Index; TST, total sleep time; NREM, non-rapid eye movement sleep; REM, rapid eye movement sleep; ODI, oxygen desaturation index; minSaO_2_, minimum oxygen saturation; T90%, percent sleep time below 90% SaO_2_; ArI, Arousal Index*.

a Compared with Primary snoring group, P < 0.05;

b *Compared with Mild-moderate OSA group, P < 0.05*.

### Power Spectral Analyses

**Figure 2:**
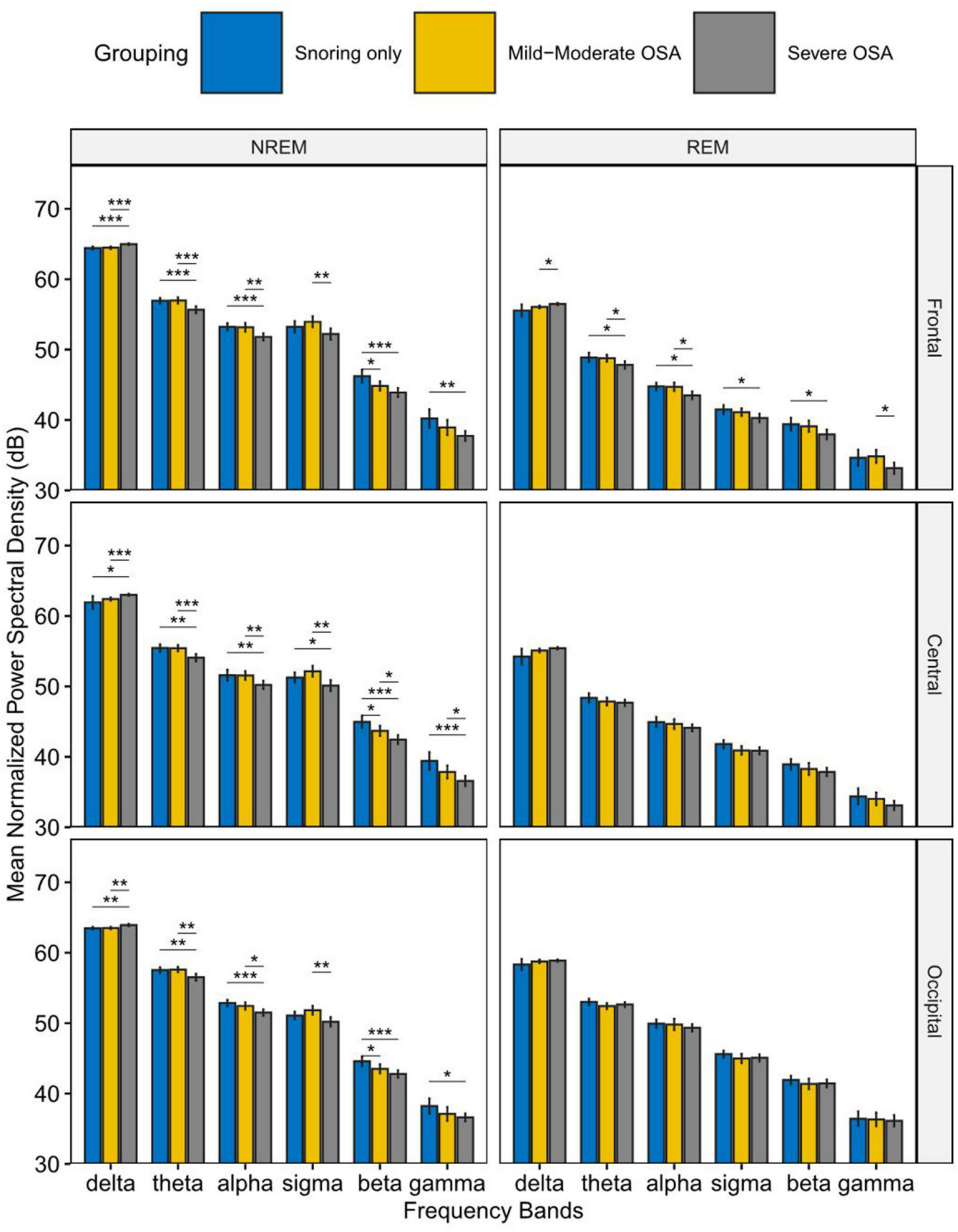
Mean Normalized Power spectral density in the primary snoring group, mild-moderate OSA group, and Severe OSA group. In NREM sleep (left), elevated normalized power spectral density (PSD) in the delta band was observed in the severe OSA group compared to the other two groups. In contrast, the PSD of the other frequency bands showed a corresponding decrease in the severe OSA group. In REM sleep (right), similar changes were observed in the frontal region. *P < 0.05, **P < 0.01, ***P < 0.001.

Figure 2 shows the normalized power spectral density among the primary snoring group, mild-moderate OSA group, and severe OSA group. In NREM sleep, elevated delta band PSD was observed in the severe OSA group compared to the other two groups. In contrast, the PSD of the other frequency bands showed a corresponding decrease in the severe OSA group, which is especially prominent in the theta and alpha frequency bands. This change was observed in the frontal, central, and occipital regions. In REM sleep, a similar pattern was observed, but only in the frontal region, i.e., an increase in delta PSD and a decrease in that of the other bands (all *p* < 0.05 and mostly *p* < 0.01).

### Relationship Between Sleep EEG Power and OSA Clinical Variables

**Figure 3:**
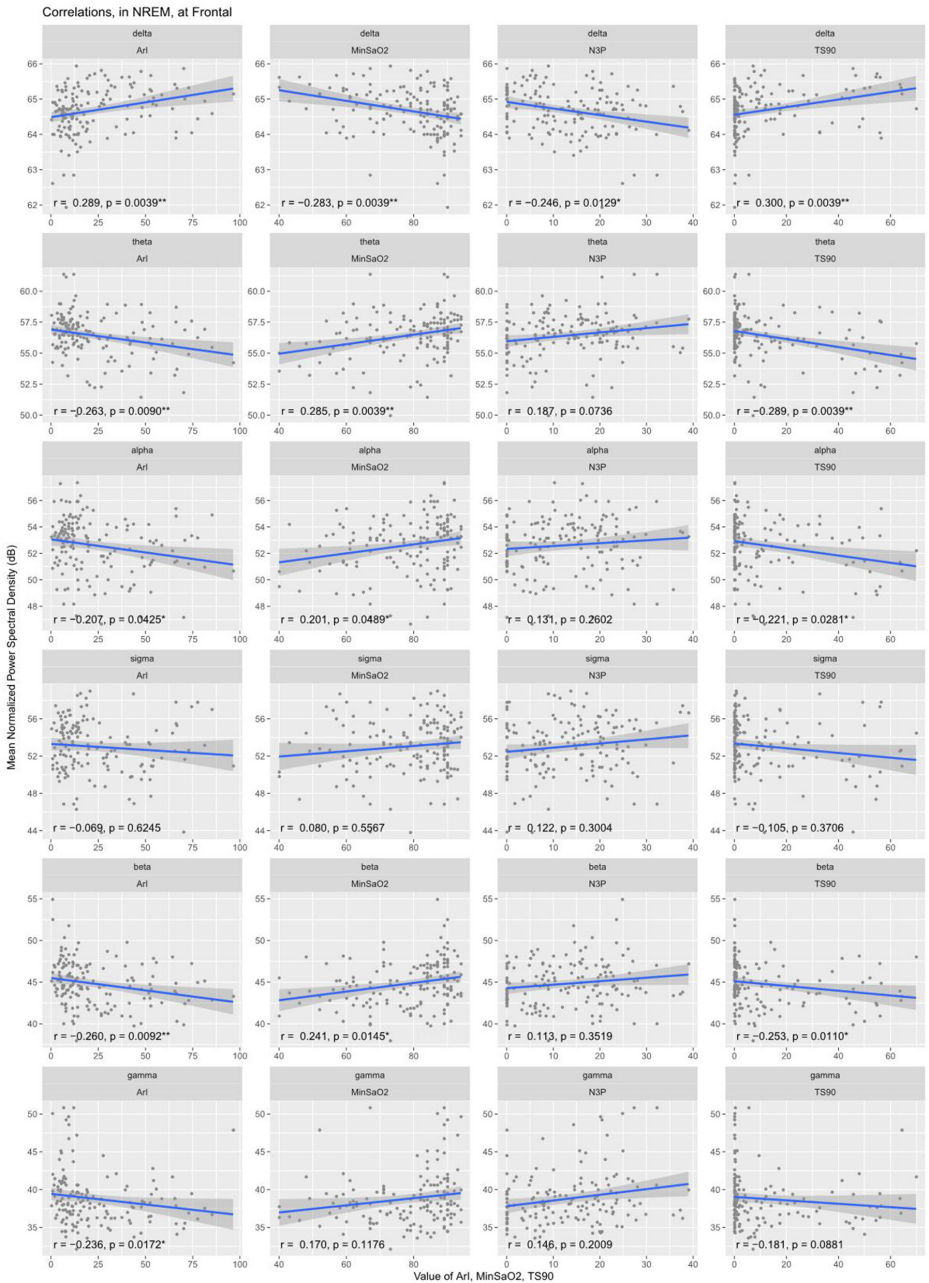
Correlation between normalized EEG spectral power density in the frontal region and clinical variables during NREM sleep. Delta band PSD was positively correlated with ArI, T90% and negatively correlated with minSaO_2_, N3%. Correspondingly, the PSD in the other frequency bands except sigma demonstrated an inverse pattern of correlations with these PSG parameters. ArI, Arousal Index; N3%, proportion of time in Stage III sleep; T90%, percent sleep time below 90% SaO_2_; minSaO_2_, minimum SaO_2_ reached during sleep.

Partial correlation analyses were conducted to explore further the associations of the EEG PSD with the clinical PSG parameters. After adjustments for patient age and corrections for multiple comparisons, delta band PSD was shown to be positively correlated with AHI (*r* = 0.33), longest time of apnea (*r* = 0.21), ODI (*r* = 0.34), T90% (*r* = 0.30), ArI (*r* = 0.29) and negatively correlated with N3% (*r* = −0.25), minSaO_2_ (*r* = −0.28) (see Table 3). Correspondingly, the PSD in the other frequency bands except sigma demonstrated an inverse pattern of correlations with these PSG parameters (see Table 3 and Figure 3).

**Table 3:** Correlation between PSG parameters and normalized power spectral density in each frequency band.

	**Delta band**	**Theta band**	**Alpha band**	**Sigma band**	**Beta band**	**Gamma band**
AHI, n/hr	0.328^**^	−0.313^**^	−0.254^*^	−0.108	−0.287^**^	−0.215^*^
TST, min	0.101	−0.026	−0.043	−0.040	−0.111	−0.162
Sleep Efficiency %	0.062	−0.077	−0.051	0.053	−0.047	−0.053
Sleep Latency, min	−0.041	0.001	−0.075	−0.011	−0.035	0.040
Longest apnea, sec	0.207^*^	−0.146	−0.249^*^	−0.075	−0.264^**^	−0.155
REM Latency, min	0.038	−0.086	−0.053	−0.125	0.023	0.120
NREM 1 %	0.134	−0.056	−0.131	−0.049	−0.124	−0.054
NREM 2 %	0.018	−0.004	0.038	−0.109	0.105	0.039
NREM 3 %	−0.246^*^	0.187	0.131	0.122	0.113	0.146
REM sleep %	0.011	0.021	0.073	0.100	−0.051	−0.145
ODI	0.342^**^	−0.320^**^	−0.241^*^	−0.112	−0.286^**^	−0.223^*^
MinSaO_2_, %	−0.283^**^	0.285^**^	0.201^*^	0.080	0.241^*^	0.170
T90 %	0.300^**^	−0.289^**^	−0.221^*^	−0.105	−0.253^*^	−0.181
ArI, n/hr	0.289^**^	−0.263^**^	−0.207^*^	−0.069	−0.260^**^	−0.236^*^
PSQI	−0.036	0.132	0.015	−0.100	0.199	0.147
ESS	0.041	−0.021	0.048	0.003	−0.011	−0.041

*The coefficients of correlation, r, are presented with statistically significant values marked by asterisks, AHI, Apnea Hypopnea Index; TST, total sleep time; NREM, non-rapid eye movement sleep; REM, rapid eye movement sleep; minSaO_2_, minimum oxygen saturation; T90%, percent sleep time below 90% SaO_2_; ArI, Arousal Index; PSQI, Pittsburgh Sleep Quality Index; ESS, Epworth Sleepiness Scale*.

* *P < 0.05*,

** *P < 0.01*.

## Discussion

This study aimed to characterize EEG power in different frequency bands during REM sleep and NREM sleep in patients with OSA. By computing the normalized spectral power densities, we found elevated normalized PSD in the delta band and decreased normalized PSD in the other frequency bands in the severe OSA group compared to the other two groups in NREM sleep. In REM sleep, similar changes were observed in the frontal region.

Two aspects distinguish this study from the previously published works on EEG spectral analysis in OSA patients: (1) previous studies have mostly focused their investigation on the EEG spectral distribution during wakefulness in OSA patients, with results showing increased low-frequency activity (delta and theta) in frontal and central regions compared with healthy controls (35, 36) and (2) few studies have so far analyzed the relative changes in the power of each frequency band in OSA patients during sleep. One previous study by Appleton et al. (37) demonstrated the absolute band spectral power in all frequency bands was higher for patients with higher AHI, and no changes were shown in the relative proportion or importance of each frequency band [see also a study by Kang et al. (38) showing similar increases in the beta and delta frequency bands].

This study thus set the focus on EEG recordings during sleep and adopted a relative measure of EEG band spectral power by normalizing the total EEG power for each participant for cross-participant comparisons, to allow a closer examination of the relative contributions by each frequency band to the total power, and how the severity of OSA mediates this change. Our results showed elevated delta power spectral density in the severe OSA group during the N2 stage compared to both the snoring only group and the mild-moderate OSA group. Correspondingly, reduced power spectral density was observed in other frequency bands in the severe OSA group compared to the other two groups. This demonstrates a shift in relative power contribution from the higher frequencies to the lower frequencies, i.e., the delta band, in the severe OSA patients. This is in agreement with previous EEG studies on OSA patients showing an increase in delta power during sleep (38, 39) and wakefulness (34). The methodology of computing normalized EEG power, however, allowed this paper to provide a more detailed analysis of the change in the relative importance of each power band, specifically, showing the relatively reduced spectral power in faster frequencies in NREM sleep, which previous studies failed to reveal.

There are a few potential explanations for this increase in delta power. In line with the two key pathophysiological mechanisms of OSA patients, hypoxia and sleep fragmentation, correlation analysis was conducted to examine whether variations in delta spectral power could be predicted by clinical variables associated with the two characteristics. For the former, hypoxia, our analysis showed that delta EEG spectral power was negatively correlated with MinSaO_2_ and positively correlated with T90%. That is, OSA patients who suffered from more severe hypoxia during the night exhibited higher spectral power in the delta frequency band. This is supported by previous studies, which demonstrated an increase in delta spectral power (40, 41) and a decrease in beta power (38) in healthy volunteers under hypoxia conditions [see however a mouse study showing contrary results (42)]. In terms of sleep fragmentation, it was found that frontal delta spectral power in NREM sleep was positively correlated with the patients' arousal index and negatively correlated with the proportion of time in N3 sleep. That is, patients with higher EEG slowing are more prone to arousals, resulting in sleep fragmentation and a reduction of deep sleep. However, it has to be cautiously noted that the evidence provided in this study is purely correlational. Future studies are required to investigate the exact causal relationship between hypoxia, sleep fragmentation, and EEG slowing.

Clinically, changes in EEG power spectral distributions and especially the EEG slowing could entail significant prognostic consequences. Studies have reported greater EEG slowing in association with poorer performance in psychomotor vigilance tasks (41) and driving simulators (43, 44). Another observational study has reported EEG slowing in frontal lateral regions in patients with memory difficulties and mild cognitive impairment (45). It is also worth noting that this increased delta band activity was observed in frontal, central, and occipital regions in the NREM stage, as was also observed previously (44). This corroborates with existing brain imaging studies demonstrating cortical activation and compromised gray and white matter integrity across multiple brain regions (frontal and parietal cortex, temporal lobe, anterior cingulate, hippocampus, and cerebellum) (46, 47). This may partly account for the wide variability of neurobehavioral disorders in OSA patients (46).

This thus suggests that the changes in slow-wave activities may be one of the pathological mechanisms of OSA. Consistent with previous studies that demonstrated pathological EEG changes in the frontal area (46), we found a significant increase in delta spectral power and a decrease in spectral power in the faster frequency bands in severe OSA patients during both NREM and REM sleep in the frontal lobe. Given that the frontal lobe is believed to be primarily responsible for regulating higher cognitive functions such as goal-directed executive function, attention, motivation, and memory, these impairments in higher cognitive functions are frequently reported in OSA patients (48). Moreover, this is made worse by the fact that the frontal lobe is more sensitive to sleep fragmentation or hypoxic damage than other brain regions (49). In our study, no apparent differences were observed among the three groups in terms of patient complaints including memory function decline, daytime sleepiness, and insomnia; neither did the PSQI questionnaires reveal any inter-group distinctions. In contrast, significant changes in the power density spectrum were detected in multiple brain regions, sensitive enough even in our set of young and middle-aged patients (39.8 ± 10.1 years). Therefore, EEG spectral analysis could be a potentially useful technique to signal early pathological brain changes in OSA patients.

There are a few limitations in this study. Our cohort consists only of young and middle-aged male patients, making our findings potentially less generalizable to the overall OSA population. Another limitation is that we chose to only analyze stage N2 among the three NREM stages, though N2 sleep is usually the most stable and it accounts for the largest proportion (~45–55%) of the total sleep time in adults. Lastly, the control group chosen in this study is primary snoring patients instead of healthy individuals, partly due to the retrospective and hospital-based nature of the study.

In summary, in OSA patients, abnormal activations of the cerebral cortex, that is, the increase in slow-wave EEG activities and the decrease in fast-wave activities, represent an increase in disease severity, degree of hypoxia, sleep fragmentation, and repeated awakening. This study also demonstrated the utility of PSA as a tool in EEG analysis in complementing the traditional PSG parameters to assess disease severity and progression.

## Data Availability Statement

The raw data supporting the conclusions of this article will be made available by the authors, without undue reservation.

## Ethics Statement

The studies involving human participants were reviewed and approved by the Research Ethics Committee of the Second Affiliated Hospital of Soochow University, Suzhou, China (JD-LK-2018-004-02). The patients/participants provided their written informed consent to participate in this study. Informed consent was sought from all patients involved, with the understanding that anonymized data could be used for research and publications.

## Author Contributions

SL collected and analyzed data and drafted the manuscript. JS designed the study, co-drafted the manuscript, was involved in the clinical diagnosis, and management of patients. YL analyzed data and co-drafted the manuscript. JinW conducted the medical assessment of the patients. QW analyzed the PSG data for the study. JX and JiaW provided guidance on experimental design and statistical analyses. RC designed experiments, participated in coordination, and critically revised the manuscript. All authors read and approved the final manuscript.

## Conflict of Interest

The authors declare that the research was conducted in the absence of any commercial or financial relationships that could be construed as a potential conflict of interest.
